# Effects of two probiotic spores of *Bacillus* species on hematological, biochemical, and inflammatory parameters in *Salmonella* Typhimurium infected rats

**DOI:** 10.1038/s41598-020-64559-3

**Published:** 2020-05-15

**Authors:** Somaye Mazkour, Seyed Shahram Shekarforoush, Sara Basiri, Saeed Nazifi, Azadeh Yektaseresht, Masoumeh Honarmand

**Affiliations:** 1 Department of Food Hygiene and Public Health, School of Veterinary Medicine, Shiraz University, Shiraz, Iran; 2 Department of Clinical Science, School of Veterinary Medicine, Shiraz University, Shiraz, Iran; 3 Department of Pathobiology, School of Veterinary Medicine, Shiraz University, Shiraz, Iran

**Keywords:** Applied microbiology, Bacterial infection

## Abstract

Salmonella infections have become a major health concern in recent decades. This pathogen has evolved to become resistant to antibiotics, which has caused problems in its treatment. As such, finding a novel preventive method is important in the treatment and management of this infection. In recent years, uses of probiotics, especially spore-former genera such as *Bacillus* spp. has become increasingly popular. In this study spores of two probiotic bacteria, *Bacillus subtilis* and *Bacillus coagulans* were fed to rats for three weeks through their daily water intake after which *Salmonella* Typhimurium was gavaged to the rats. On days 1, 3, 5 and 7 after gavaging, the number of Salmonella was counted in liver, spleen, mesenteric lymph nodes, feces and content of ileum and cecum. Hematological and biochemical parameters, inflammatory mediators, total antioxidant capacity and malondialdehyde were also measured. The results showed that *B. subtilis* and *B. coagulans* caused delation in infiltration of Salmonella into the lymph nodes, spleen and liver, reduction of the inflammatory mediators, and decreases in oxidative stress, hematological and biochemical changes. The overall count of Salmonella in the above mentioned parameters has also decreased and a faster return to normal base were also witnessed. The results showed that the use of *B. subtilis* and *B. coagulans* can potentially help boost the body’s immune system, to combat the effects of exposure to the Salmonella pathogen.

## Introduction

Salmonella infections are considered to be a serious and harmful infection affecting humans and animals and a great concern for human health^1^. Direct contact with infected animals or their products makes humans infected. Among *Salmonella* spp., *Salmonella enterica* serovar Enteritidis (*S*. Enteritidis) and *Salmonella enterica* serovar Typhimurium (*S*. Typhimurium) are the main serovars that cause human salmonellosis^2^. Salmonellosis causes a lot of costs, which includes treatment costs, productivity reduction, the value of premature death and also economic losses because of the livestock and poultry death^3^. Preventative measures such as usage of probiotics in animals can control and reduce Salmonella infection, and its costs^4,5^.

Probiotics are live microorganisms, which have health benefits if administered in adequate amounts^6,7^. Most of the reported research on probiotics have focused on the various strains of *Lactobacillus*. In recent years, uses of spore-forming bacteria and especially *Bacillus* spp., as probiotics have increased^8^. The bacteria’s ability to form spore results in higher resistance during the production and storage processes, as well as gastric (pH, digestive enzymes) and intestinal environmental conditions^9^. Through direct contact with epithelial cells, also immune cells, activation of gut microflora and modification of its composition, innate and adaptive immunity are influenced by probiotics^10^. Modulation of pro-inflammatory (like IFN-γ and TNF-α) and anti-inflammatory cytokines (IL-10) is one of the principal mechanisms that probiotic influence the immune system^11^.

Many studies have investigated the effect of spore-forming probiotics such as *Bacillus* spp. and non spore-forming probiotics such as Lactobacilli on Salmonella infection and most found that probiotics reduce the Salmonella-caused injuries such as gut inflammation, serological factors enhancement, hematological changes, and even the number of Salmonella in target organs and intestine^4,12–15^. Some studies tested a combination of Lactobacilli probiotics^5,16^.

Among the known *Bacillus* spp., solely *Bacillus coagulans* and *Bacillus subtilis* var. *natto* have been accepted in general for human consumption. Some of these species’ beneficial have been examined individually. For example, *B. subtilis* var. *natto* stimulates the immune system, has anti-cancer properties and produces vitamin K_2_, and *B. coagulans* is an aid for the absorption and consumption of proteins^17–19^. However, there is no study investigating the combined effect of *Bacillus* spp. on Salmonella infection. So, the current study was conducted to evaluate the possible effect of the combination of *B. subtilis* and *B. coagulans* on hematological, biochemical, inflammatory mediators and oxidation parameters, and also the number of Salmonella in the intestine and affected organs in experimentally Salmonella-infected rats. It was expected that the combined administration of *B. subtilis* and *B. coagulans* would cause the inflammation reduction through the decrease of the inflammatory markers.

## Results and Discussion

### Enumeration of Salmonella

To find out the *B. subtilis* and *B. coagulans* effects on Salmonella count in digestive tract and its infiltration to the target organs, the number of *S*. Typhimurium in the liver, spleen, mesenteric lymph nodes, feces, and content of ileum and cecum was counted on days 1, 3, 5 and 7 after gavaging Salmonella. The results are shown in Tables 1 and 2 in detail. No Salmonella was isolated from the Blank Control and Probiotic Control groups on all sampling days. On the first day of sampling, by direct plating, the number of Salmonella in the liver, spleen and mesenteric lymph nodes of all four treatment groups was zero. But by using selective enrichment, lymph nodes of four rats and the liver of one rat from the Salmonella group, and lymph nodes of one rat from the Probiotic+Salmonella group showed the presence of Salmonella. On the 3^rd^, 5^th^ and 7^th^ days post gavaging, Salmonella was countable in the liver, spleen and lymph nodes of Salmonella and Probiotic+Salmonella groups. The results show that the number of Salmonella is higher in the lymph nodes than in the liver and spleen (*P* < 0.05). Using probiotics, the number of Salmonella in the liver, spleen and mesenteric lymph nodes was significantly reduced in all three days of sampling(*P* < 0.05); also, the results show that over time, the reduction trend in Salmonella increased. The number of Salmonella excreted in the ileum, cecum and feces increased until the 5^th^ day post gavaging and decreased again on the 7^th^ day, which was similar in Salmonella and Probiotic+Salmonella groups. However, the number of Salmonella in the ileum (*P* < 0.05), cecum (*P* < 0.05) and feces in Probiotic+Salmonella group was less than the Salmonella group from the first day of sampling.

**Table 1:** Number of Salmonella (Log CFU/gr) in liver, spleen and mesenteric lymph nodes of rats with different treatments in 1, 3, 5 and 7 days after gavaging Salmonella.

Groups	Liver				Spleen				Mesenteric lymph nodes			
	1^st^ day	3^rd^ day	5^th^ day	7^th^ day	1^st^ day	3^rd^ day	5^th^ day	7^th^ day	1^st^ day	3^rd^ day	5^th^ day	7^th^ day
Probiotic+Salmonella	0.0 ± 0.0^aA^	2.4 ± 0.3^cB^	2.5 ± 0.4^cB^	1.5 ± 0.9^cC^	0.0 ± 0.0^aA^	2.4 ± 0.2^cB^	2.8 ± 0.4^cB^	2.4 ± 0.6^cB^	0.0 ± 0.0^aA^	4.2 ± 0.2^cBC^	4.7 ± 0.3^cC^	3.9 ± 0.8^cB^
Blank Control	0.0 ± 0.0^aA^	0.0 ± 0.0^aA^	0.0 ± 0.0^aA^	0.0 ± 0.0^aA^	0.0 ± 0.0^aA^	0.0 ± 0.0^aA^	0.0 ± 0.0^aA^	0.0 ± 0.0^aA^	0.0 ± 0.0^aA^	0.0 ± 0.0^aA^	0.0 ± 0.0^aA^	0.0 ± 0.0^aA^
Probiotic Control	0.0 ± 0.0^aA^	0.0 ± 0.0^aA^	0.0 ± 0.0^aA^	0.0 ± 0.0^aA^	0.0 ± 0.0^aA^	0.0 ± 0.0^aA^	0.0 ± 0.0^aA^	0.0 ± 0.0^aA^	0.0 ± 0.0^aA^	0.0 ± 0.0^aA^	0.0 ± 0.0^aA^	0.0 ± 0.0^aA^
Salmonella	0.0 ± 0.0^aA^	2.9 ± 0.2^bB^	3.3 ± 0.5^bB^	2.9 ± 0.5^bB^	0.0 ± 0.0^aA^	2.8 ± 0.4^bB^	3.4 ± 0.2^bC^	3.1 ± 0.1^bBC^	0.0 ± 0.0^aA^	4.6 ± 0.2^bB^	5.1 ± 0.1^bC^	4.7 ± 0.3^bB^

Values are mean ± standard deviation of 5 independent replicates. The different small letters in columns indicate significant differences among groups (P < 0.05). The different capital letters in each row for each parameter indicate significant differences among post infectious sampling days (P < 0.05).

**Table 2:** Number of Salmonella (Log CFU/gr) in the ileum, cecum and feces of rats with different treatments in 1, 3, 5 and 7 days after gavaging Salmonella.

Groups	Ileum				Cecum				Feces			
	1^st^ day	3^rd^ day	5^th^ day	7^th^ day	1^st^ day	3^rd^ day	5^th^ day	7^th^ day	1^st^ day	3^rd^ day	5^th^ day	7^th^ day
Probiotic+Salmonella	4.4 ± 0.2^cA^	5.4 ± 0.1^cB^	6.7 ± 0.1^cC^	4.7 ± 0.2^cD^	4.8 ± 0.2^cA^	5.4 ± 0.1^cB^	6.5 ± 0.3^cC^	4.6 ± 0.3^cA^	5.1 ± 0.2^cA^	5.3 ± 0.2^bA^	6.5 ± 0.2^bB^	4.5 ± 0.6^cC^
Blank Control	0.0 ± 0.0^aA^	0.0 ± 0.0^aA^	0.0 ± 0.0^aA^	0.0 ± 0.0^aA^	0.0 ± 0.0^aA^	0.0 ± 0.0^aA^	0.0 ± 0.0 ^aA^	0.0 ± 0.0 ^aA^	0.0 ± 0.0 ^aA^	0.0 ± 0.0 ^aA^	0.0 ± 0.0 ^aA^	0.0 ± 0.0 ^aA^
Probiotic Control	0.0 ± 0.0^aA^	0.0 ± 0.0^aA^	0.0 ± 0.0^aA^	0.0 ± 0.0^aA^	0.0 ± 0.0^aA^	0.0 ± 0.0^aA^	0.0 ± 0.0 ^aA^	0.0 ± 0.0 ^aA^	0.0 ± 0.0 ^aA^	0.0 ± 0.0 ^aA^	0.0 ± 0.0 ^aA^	0.0 ± 0.0 ^aA^
Salmonella	5.3 ± 0.2^bA^	5.6 ± 0.1^bA^	6.8 ± 0.1^bB^	5.4 ± 0.2^bA^	5.2 ± 0.3^bA^	5.6 ± 0.1^bB^	6.8 ± 0.2^bC^	5.3 ± 0.4^bAB^	5.6 ± 0.3^bA^	5.5 ± 0.3^bA^	6.6 ± 0.4^bB^	5.0 ± 0.3^bC^

Values are mean ± standard deviation of 5 independent replicates. The different small letters in columns indicate significant differences among groups (P < 0.05). The different capital letters in each row for each parameter indicate significant differences among post infectious sampling days (P < 0.05).

The correlation coefficient between the number of Salmonella in the ileum, cecum and feces with the number of Salmonella in the liver was 0.67, 0.54 and 0.69; in the spleen was 0.72, 0.83 and 0.71; and in the mesenteric lymph nodes was 0.72, 0.78 and 0.66, respectively (*P* < 0.05).

As the results show, the probiotics *B. subtilis* and *B. coagulans* did not completely inhibit the infiltration of Salmonella into lymph nodes, but they caused delayed Salmonella infiltration into lymph nodes and consequently into spleen and liver. Additionally, the probiotics accelerate the deletion of this pathogen in the infected group. As previous studies have shown, probiotics have the beneficial effect of preventing the colonization of pathogenic bacteria in the digestive tract, competition for nutrients and adhesion receptors, and stimulation of host immunity^20,21^. As the number of Salmonella was at the highest levels on the 5^th^ day post gavaging and the differences between the two groups, it can be said that *B. subtilis* and *B. coagulans* reduce the *S*. Typhimurium colonization and invasion. Vila *et al*. (2009) also showed that *B. cereus* var. *toyoi* decrease the colonization and invasion of *S*. Entritidis in the broiler^22^. La Ragione and Woodward (2003) showed that gavaging *B. subtilis* even one day before receiving *S*. Entertidis, reduced the number of Salmonella in the liver, spleen, jejenum, ileum, colon and cecum. The number of *S*. Enteritidis recovered from caeca of chicks that had received a pre-dose of *B. subtilis* spores wassignificantly lower than the control group that had not received *B. subtilis*^12^. Feng *et al*. (2016) showed that *Lactobacillus plantarum* PZ01, *Lactobacillus salivarius* JM32 and *Pediococcus acidilactici* JH231 reduced the number of Salmonella in intestinal content, spleen and liver during a Salmonella infection in Broiler Chicks^5^. Besides that, Barba-Vidal *et al*. (2017) showed that the administration of *Bifidobacterium longum* subsp. *infantis* CECT 7210 and *Bifidobacterium animalis* subsp. *lactis* BPL6 in a weaning piglet model was not able to prevent the Salmonella infection, as most all of them became positive in the feces one day after the challenge. Despite this, the probiotic was able to reduce the pathogen load in the colon and feces, suggesting the potential of the bifidobacteria combination to exclude Salmonella^23^.

### Hematological parameters

The results showed that the packed cell volume (PCV) percentage, red blood cell (RBC) count (until the 5^th^ day) and hemoglobin (Hb) concentration (until the 3^rd^ day) were greater in the Salmonella group than the other groups (*P* < 0.05). While, these values returned to the normal range by day 7 (Table 3). These increments were a result of severe dehydration in the Salmonella group, which occurred due to diarrhea^22^. In Salmonella group, the stool was looser than the Blank Control group for 3 days after gavaging. Previous studies have shown that the *Salmonella* Typhimurium increase the Hb, PCV and RBC amount of piglets^23^ and calves^24^. In the Salmonella group the RBC count had a decreasing trend from the 1^st^ day to the 7^th^ day of infection (*P* < 0.05). However, in the Probiotic+Salmonella group there was no increase in the PCV, RBC count and Hb concentration and these parameters were similar to the Blank Control group. In Probiotic+Salmonella group, Salmonella showed lower pathogenicity due to the presence of active probiotics that prevented the colonization of Salmonella and competed for nutrients and adhesion receptors^20,21^.

**Table 3:** The PCV, Hb and RBC of blood samples of rats with different treatments in 1, 3, 5 and 7 days after gavaging Salmonella.

Groups	PCV (%)				Hb (gr/dL)				RBC (10^6^/μL)			
	1^st^ day	3^rd^ day	5^th^ day	7^th^ day	1^st^ day	3^rd^ day	5^th^ day	7^th^ day	1^st^ day	3^rd^ day	5^th^ day	7^th^ day
Probiotic+Salmonella	48.8 ±1.5 ^aA^	47.0 ±0.8 ^aA^	47.4 ±1.0 ^aA^	48.2 ±2.2 ^aA^	15.8 ±0.9^abA^	15.1 ±0.5 ^aA^	15.1 ±1.0 ^aA^	15.1 ±0.7 ^aA^	9.0 ±0.3 ^aA^	8.4 ±0.3^aAB^	8.2 ±0.7^aB^	8.4 ±0.3^aAB^
Blank Control	47.5 ±0.7^aA^	47.0 ±1.1 ^aA^	48.1 ±0.6 ^aA^	47.2 ±1.8 ^aA^	15.5 ±0.6 ^aA^	14.8 ±0.4 ^aA^	15.0 ±0.4 ^aA^	15.2 ±0.7 ^aA^	8.4 ±0.4 ^aA^	8.3 ±0.5 ^aA^	8.3 ±0.2 ^aA^	8.5 ±0.6 ^aA^
Probiotic Control	48.2 ±2.1^aA^	47.8 ±0.5 ^aA^	47.9 ±0.5 ^aA^	46.9 ±2.4 ^aA^	15.4 ±0.3 ^aA^	14.9 ±0.2 ^aA^	15.4 ±0.5 ^aA^	15.8 ±1.3 ^aA^	8.6 ±0.3 ^aA^	8.5 ±0.3 ^aA^	8.4 ±0.3 ^aA^	8.5 ±0.4 ^aA^
Salmonella	51.1 ±1.2^bA^	49.4 ±1.3^bB^	49.2 ±0.5^bB^	47.2 ±1.5 ^aC^	16.4 ±0.3^bA^	16.1 ±0.3^bA^	15.9 ±0.5 ^aA^	15.1 ±0.6^aB^	9.5 ±0.4^bA^	9.2 ±0.2^bAB^	8.9 ±0.4^bB^	8.4 ±0.4^aC^

Values are mean ± standard deviation of 5 independent replicates. The different small letters in columns indicate significant differences among groups (P < 0.05). The different capital letters in each row for each parameter indicate significant differences among post infectious sampling days (P < 0.05).

The results show that no significant difference was observed in platelet counts (*P* > 0.05) (Data not shown).

The number of white blood cell (WBC) and neutrophils in the Salmonella group was significantly higher than the other groups from the first day after gavaging. The WBC and neutrophils count were increased during three days of infection and then started to come down from day 5 onwards. The use of probiotics before gavaging Salmonella, reduced the increasing trend of the WBC and neutrophils levels and eventually brought up the levels of these two indicators to the normal range by day 5. On day 7, there was no significant differences in the number of WBC and neutrophils between Salmonella and Probiotic+Salmonella groups (*P* > 0.05). The counts were greater than Blank Control and Probiotic Control groups (Table 4).

**Table 4:** The haematological parameters of blood samples of rats with different treatments in 1, 3, 5 and 7 days after gavaging Salmonella.

Groups	WBC (10^4^/μL)				Neutrophile (10^3^/ μL)				Lymphocyte (10^3^/μL)			
	1^st^ day	3^rd^ day	5^th^ day	7^th^ day	1^st^ day	3^rd^ day	5^th^ day	7^th^ day	1^st^ day	3^rd^ day	5^th^ day	7^th^ day
Probiotic+Salmonella	1.38 ±0.21 ^aA^	1.46 ±0.17 ^aA^	1.49 ±0.21^cA^	1.43 ±0.09^bA^	3.87 ±0.47^cA^	3.85 ±0.40 ^aA^	3.57 ±0.57^bA^	3.86 ±0.25^cA^	9.11 ±1.56 ^aA^	9.91 ±1.32 ^aA^	10.80 ±1.76^bA^	9.96 ±0.59 ^aA^
Blank Control	1.14 ±0.06 ^aA^	1.23 ±0.06 ^aA^	1.17 ±0.10 ^aA^	1.17 ±0.11 ^aA^	3.16 ±0.20 ^aA^	2.72 ±0.28^aAB^	2.47 ±0.53^aAB^	2.00 ±0.85^aB^	7.76 ±0.42 ^aA^	8.90 ±0.35^aAB^	8.41 ±1.39^aAB^	9.18 ±0.75 ^aB^
Probiotic Control	1.17 ±0.08 ^aA^	1.14 ±0.03 ^aA^	1.01 ±0.14^aB^	1.15 ±0.04 ^aA^	3.00 ±0.39 ^aA^	3.18 ±0.23 ^aA^	2.85 ±0.52^abA^	3.03 ±0.26^bA^	7.91 ±0.62 ^aA^	7.47 ±0.58^aAB^	6.69 ±1.01^aB^	7.47 ±0.37^bAB^
Salmonella	1.73 ±0.35^bA^	2.02 ±0.63^bA^	1.78 ±0.15^bA^	1.51 ±0.12^bA^	4.64 ±0.73^bA^	5.57 ±1.71^bA^	4.94 ±0.80^cA^	4.15 ±0.61^cA^	11.70 ±2.40^bA^	14.00 ±5.31^bA^	12.10 ±0.84^bA^	10.60 ±0.83^cA^

Values are mean ± standard deviation of 5 independent replicates. The different small letters in columns indicate significant differences among groups (P < 0.05). The different capital letters in each row for each parameter indicate significant differences among post infectious sampling days (P < 0.05).

The introduction of Salmonella significantly increased the number of lymphocytes compared to the Blank Control group (*P* < 0.05). While using probiotics before gavaging Salmonella, significantly reduced the increasing trend of lymphocytes (*P* < 0.05). These results held true from days 1 to 5 but on the 5^th^ day, no significant difference between Salmonella and Probiotic+Salmonella groups (*P* > 0.05) were observed. On the 7^th^ day, the number of lymphocyte in Probiotic+Salmonella group returned to normal levels and there were no differences in levels compared to the Blank Control group. However, the Salmonellal group had greater levels of lymphocytes compared to the other three groups (*P* < 0.05) (Table 4).

No significant differences were observed in the levels of monocytes, eosinophils and basophils among the four treatment groups (*P* > 0.05) (Data not shown).

The correlation coefficient between the number of Salmonella in the ileum, cecum, feces, liver, spleen and mesenteric lymph nodes with the number of WBC was 0.32, 0.36, 0.30, 0.33 and 0.47 and 0.48; neutrophile was 0.26, 0.32, 0.24, 0.29, 0.47 and 0.47; and lymphocyte was 0.32, 0.36, 0.30, 0.30, 0.44 and 0.45, respectively (*P* < 0.05).

In this experimental study, the number of WBC in the Salmonella group was significantly more than other groups from the first day after gavaging due to the increase in neutrophiles and lymphocytes. This was also confirmed before that Salmonella caused neutrophile and lymphocyte increases resulted in the WBC count increase^24,25^. Also, the increase in the number of Salmonella in the ileum, cecum, feces, liver, spleen, and mesenteric lymph nodes were accompanied by an increase in the number of WBC, neutrophils and lymphocytes (*P* < 0.05). However, in the Probiotic+Salmonella group, these levels rose after a delay. These results would indicate that *B. subtilis* and *B. coagulans* decrease the effects of *S*. Typhimurium. Additionally, in the Probiotic+Salmonella group, the complications from *S*. Typhimurium are seen in a shorter period of time and the changes returned to the normal base quicker than the Salmonella group. Havelaar *et al*. (2001) showed that *S*. Enteritidis in rats cause detectable changes in neutrophile counts from doses of 10^4^ CFU upwards and large increases in WBC and neutrophils levels were observed after five days. These results showed that all leukocyte types, except eosinophils, have positive dose response relationships^26^.

### Biochemical parameters

There was no significant difference in biochemical parameters between Blank Control and Probiotic Control groups (*P* > 0.05). The Salmonella infection increased the total protein and globulin amounts in the blood (*P* < 0.05), but had no significant effect on blood serum albumin (*P* > 0.05). Consumption of probiotics in Salmonella group, significantly reduced the total protein and globulin levels (*P* < 0.05) and brought these levels close to the control values (Table 5). The amount of globulin and total protein on the 1^st^, 3^rd^ and 5^th^ days was significantly higher in the Salmonella group compared to the Probiotic+Salmonella group, and both were higher than the Blank Control group. On the 7^th^ day with Probiotic+Salmonella group, the amount of globulin and total protein was similar to the Blank Control group. Oral inoculation of Salmonella, stimulates the immune responses, including humoral and cell-mediated responses, which increases the serum level of immunoglobulin and leads to an increase in total protein^27^. As the results show, no differences were observed in the amount of albumin among the groups. Therefore, the changes in the total protein are due to the change in the amount of globin levels. The increase in the number of Salmonella in the liver, spleen and mesenteric lymph nodes were accompanied by serum globulin and total protein increases (*P* < 0.05). The correlation coefficient between the number of Salmonella in the liver, spleen and mesenteric lymph nodes with the amount of total protein of blood was 0.25, 0.28 and 0.35; globulin was 0.25, 0.35 and 0.43, respectively. The *B. subtilis* and *B. coagulans* reduce the globulin levels and also the total protein levels in comparison to the Salmonella group. This indicates that *B. subtilis* and *B. coagulans* reduce the pathogenic effect of *S*. Typhimurium. Abudabos *et al*. (2016) showed that Bacillus as probiotic had no effect on total protein, globulin and albumin levels. When exposed to Salmonella, probiotics inhibit albumin decreases and globulin increases which resulted in positive control of Salmonella^28^.

**Table 5:** The biochemical parameters of blood samples of rats with different treatments in 1, 3, 5 and 7 days after gavaging Salmonella.

Groups	Total protein (gr/dL)				Albumin (gr/dL)				Globulin (gr/dL)			
	1^st^ day	3^rd^ day	5^th^ day	7^th^ day	1^st^ day	3^rd^ day	5^th^ day	7^th^ day	1^st^ day	3^rd^ day	5^th^ day	7^th^ day
Probiotic+Salmonella	7.1 ±0.4^cA^	6.8 ±0.3^aAB^	6.8 ±0.2^cAB^	6.6 ±0.2^aB^	3.6 ±0.2 ^aA^	3.3 ±0.2^aB^	3.5 ±0.1^aAB^	3.5 ±0.1^aAB^	3.5 ±0.4^cA^	3.5 ±0.1^cA^	3.2 ±0.2^cB^	3.1 ±0.1^aB^
Blank Control	6.6 ±0.2 ^aA^	6.3 ±0.5 ^aA^	6.5 ±0.2^abA^	6.7 ±0.2 ^aA^	3.8 ±0.1 ^aA^	3.4 ±0.3^aB^	3.6 ±0.2 ^aAB^	3.7 ±0.2^aA^	2.9 ±0.1 ^aA^	2.9 ±0.2 ^aA^	3.0 ±0.2 ^aA^	3.0 ±0.1 ^aA^
Probiotic Control	6.7 ±0.2 ^aA^	6.4 ±0.2 ^aA^	6.3 ±0.2 ^aA^	6.3 ±0.7 ^aA^	3.7 ±0.2 ^aA^	3.5 ±0.2 ^aA^	3.4 ±0.1 ^aA^	3.5 ±0.4 ^aA^	3.0 ±0.1 ^aA^	2.9 ±0.1 ^aA^	2.9 ±0.2 ^aA^	2.8 ±0.3 ^aA^
Salmonella	7.6 ±0.2^bA^	7.8 ±0.8^bA^	7.2 ±0.3^bA^	7.2 ±0.3^bA^	3.6 ±0.3 ^aA^	3.5 ±0.2 ^aA^	3.4 ±0.2 ^aA^	3.6 ±0.2 ^aA^	4.1 ±0.3^bA^	4.3 ±0.9^bA^	3.8 ±0.3^bA^	3.6 ±0.3^bA^

Values are mean ± standard deviation of 5 independent replicates. The different small letters in columns indicate significant differences among groups (P < 0.05). The different capital letters in each row for each parameter indicate significant differences among post infectious sampling days (P < 0.05).

There was no significant difference in the amount of alkaline phosphatase (ALP) between four groups during the sampling days (*P* > 0.05) (Data not shown). No significant differences were observed in the amount of alanine transaminase (ALT) and aspartate transaminase (AST) among four groups until the 5^t^^h^ day of sampling (*P* > 0.05), while, on the 7^th^ day, Salmonella caused a significant increase in the ALT and AST activity (*P* < 0.05). Probiotic consumption kept the level of these two enzymes in the normal range throughout the sampling period (Table 6). Increase in ALT and AST on the 7^th^ day post oral inoculation in Salmonella group, indicate the inflammation and damage in the liver^29^. However, in the Probiotic+Salmonella group, no increase was observed, which means *B. subtilis* and *B. coagulans* inhibit the pathogenic effect of *S*. Typhimurium and inflammation in the liver. Also lesser changes in hepatic factor in Probiotic+Salmonella group is due to the lesser count of Salmonella in this group in comparison to Salmonella group.

**Table 6:** The haematological parameters of blood samples of rats with different treatments in 1, 3, 5 and 7 days after gavaging Salmonella.

Groups	ALT (U/L)				AST (U/L)				Glucose (mg/dL)			
	1^st^ day	3^rd^ day	5^th^ day	7^th^ day	1^st^ day	3^rd^ day	5^th^ day	7^th^ day	1^st^ day	3^rd^ day	5^th^ day	7^th^ day
Probiotic+Salmonella	46.1 ±9.3 ^aA^	50.8 ±10.9 ^aA^	54.3 ±7.5 ^aA^	56.2 ±10.4 ^aA^	156.0 ±41.3 ^aA^	162.6 ±40.1 ^aA^	187.0 ±8.6 ^aA^	166.0 ±47.5 ^aA^	95.8 ±6.3 ^aA^	91.7 ±5.6 ^aA^	84.4 ±15.6 ^aA^	88.9 ±4.0 ^aA^
Blank Control	48.9 ±7.4 ^aA^	52.8 ±13.0 ^aA^	51.0 ±7.1 ^aA^	51.0 ±4.4 ^aA^	186.6 ±12.5 ^aA^	201.6 ±39.8 ^aA^	205.6 ±32.7 ^aA^	177.8 ±26.6 ^aA^	90.4 ±15.7 ^aA^	84.9 ±11.0 ^aA^	91.4 ±12.5 ^aA^	93.9 ±2.7 ^aA^
Probiotic Control	52.5 ±4.3 ^aA^	51.3 ±18.8 ^aA^	50.9 ±15.8 ^aA^	51.0 ±11.1 ^aA^	167.2 ±41.8 ^aA^	185.2 ±43.9 ^aA^	176.0 ±17.5 ^aA^	168.2 ±61.2 ^aA^	96.6 ±6.8^aB^	88.2 ±10.2^aAB^	75.3 ±14.5 ^aA^	93.8 ±8.6^aB^
Salmonella	52.1 ±4.3 ^aA^	56.7 ±17.5 ^aA^	54.1 ±2.3 ^aA^	78.1 ±6.3^bB^	182.4 ±52.8 ^aA^	184.4 ±23.3 ^aA^	162.4 ±50.6 ^aA^	261.4 ±25.5 ^bB^	111.8 ±4.6^**bA**^	107.8 ±4.1^bA^	78.4 ±6.8^aB^	86.8 ±5.5^aB^

Values are mean ± standard deviation of 5 independent replicates. The different small letters in columns indicate significant differences among groups (P < 0.05). The different capital letters in each row for each parameter indicate significant differences among post infectious sampling days (P < 0.05).

During infections, *S*. Typhimurium disseminates systemically from the Peyer’s patches to the liver and spleen, where it continues to grow within macrophages^30,31^. Glycolysis is required for intracellular replication and survival of *S*. Typhimurium in macrophages^32^. The lower glucose could be due to a greater blood glucose uptake when the metabolism is responding against the Salmonella infection^23^. In the Salmonella group, the glucose amounts increased on the 1^st^ and 3^rd^ days post inoculation (Table 6), but stayed within the normal range in the Probiotic+Salmonella group, which means *B. subtilis*, and *B. coagulans* reduce the effects of Salmonella. The increase in the number of Salmonella in the ileum, cecum, feces, liver and spleen was accompanied with serum glucose reduction (*P* < 0.05). The correlation coefficient between the number of Salmonella in the ileum, cecum, feces, liver and spleen with the serum glucose was -0.28, -0.31, -0.26, -0.22 and -0.22 in order (*P* < 0.05).

The Salmonella infection increased the level of lactate dehydrogenase (LDH) significantly (*P* < 0.05). LDH is recognized as a marker for cell toxicity and the lower amount indicates lower cell injuries^33^. The use of probiotics in Salmonella infected rats, lead to smaller increment in the LDH level, however, it was still higher than the Blank Control and Probiotic Control groups (*P* < 0.05) (Table 7). This indicates that *B. subtilis* and *B. coagulans* decrease the cell injuries resulted by Salmonella. The increase in the number of Salmonella in the ileum, cecum, feces, liver, spleen, and mesenteric lymph nodes were accompanied by LDH increases (*P* < 0.05).

**Table 7:** The biochemical parameters of blood samples of rats with different treatments in 1, 3, 5 and 7 days after gavaging Salmonella.

Groups	LDH (U/L)				Creatine (mg/dL)				BUN (mg/dL)				Total iron (µg/dL)			
	1^st^ day	3^rd^ day	5^th^ day	7^th^ day	1^st^ day	3^rd^ day	5^th^ day	7^th^ day	1^st^ day	3^rd^ day	5^th^ day	7^th^ day	1^st^ day	3^rd^ day	5^th^ day	7^th^ day
Probiotic+Salmonella	2471.6 ±430.2 ^aA^	2643.8 ±222.9^cA^	2990.6 ±495.4^cA^	3901.2 ±313.7^cB^	0.52 ±0.07 ^aA^	0.62 ±0.09 ^aA^	0.57 ±0.07 ^aA^	0.55 ±0.09 ^aA^	41.0 ±3.0 ^aA^	43.3 ±4.9 ^aA^	43.1 ±4.8 ^aA^	43.2 ±5.1 ^aA^	328.9 ±93.0 ^bA^	196.0 ±49.8 ^aB^	202.4 ±68.8^aB^	153.0 ±22.6^aB^
Blank Control	2194.0 ±167.5^aA^	2243.2 ±360.0 ^aA^	2131.2 ±157.7 ^aA^	2402.0 ±244.8 ^aA^	0.61 ±0.03 ^aA^	0.60 ±0.12 ^aA^	0.59 ±0.04 ^aA^	0.61 ±0.08 ^aA^	42.9 ±4.8 ^aA^	43.0 ±5.8 ^aA^	44.4 ±4.3 ^aA^	46.5 ±4.6 ^aA^	155.2 ±30.5 ^aA^	159.2 ±26.5^aAB^	199.0 ±32.8^aB^	162.0 ±22.9^aAB^
Probiotic Control	2063.6 ±374.2 ^aA^	2216.8 ±109.9 ^aA^	2111.2 ±491.9 ^aA^	2140.4 ±509.8 ^aA^	0.58 ±0.04 ^aA^	0.52 ±0.03 ^aA^	0.58 ±0.07 ^aA^	0.58 ±0.14 ^aA^	44.9 ±5.3 ^aA^	40.3 ±4.9 ^aA^	44.9 ±9.9 ^aA^	45.3 ±5.1 ^aA^	151.0 ±46.1 ^aA^	166.4 ±31.4 ^aA^	140.0 ±17.3 ^aA^	154.5 ±72.3 ^aA^
Salmonella	3148.2 ±588.7^bA^	3231.4 ±328.0^bA^	4580.4 ±916.9^bB^	5628.0 ±1035.2^bC^	0.57 ±0.11 ^aA^	0.62 ±0.13 ^aA^	0.72 ±0.08^bA^	0.72 ±0.13^bA^	43.8 ±6.9 ^aA^	44.8 ±6.1 ^aA^	59.4 ±3.6^bB^	54.4 ±2.7^bB^	345.2 ±48.0^bA^	497.2 ±93.9^bB^	281.8 ±85.3^bAC^	228.4 ±92.6^aC^

Values are mean ± standard deviation of 5 independent replicates. The different small letters in columns indicate significant differences among groups (*P* < 0.05). The different capital letters in each row for each parameter indicate significant differences among post infectious sampling days (*P* < 0.05).

In the Salmonella group, creatine levels increased from the 5^th^ day of gavaging. The same trend was seen in the blood urea nitrogen (BUN) on the 5^th^ and 7^th^ days of sampling, which indicated kidney dysfunction. It was also mentioned by Doorn *et al*. (2006) that in Salmonella infection, renal dysfunction occurred and this causes BNU and Creatinin increases^34^. In the Probiotic+Salmonella group, the amount of creatine and BUN were similar to the Blank Control and Probiotic Control groups (*P* > 0.05) on all sampling days (Table 7). It indicates that *B. subtilis* and *B. coagulans* inhibit the pathogenic effect of *S*. Typhimurium on kidneys.

The increase in the number of Salmonella in the liver, spleen and mesenteric lymph nodes were accompanied with BUN and creatine increases (*P* < 0.05). The correlation coefficient between the number of Salmonella in the liver, spleen and mesenteric lymph nodes with the amount of creatine was 0.28, 0.40 and 0.45; and BUN was 0.41, 0.59 and 0.53, respectively (*P* < 0.05).

The level of total iron in the Salmonella group increased significantly on days 1, 3 and 5, and then returned to the normal range on the 7^th^ day. *S*. Typhimurium secretes siderophores to bind to the ferric iron^35^. However the use of probiotics maintained the total blood iron level within normal range in all sampling days (Table 7). In the Probiotic+Salmonella group, it has been higher just on the 1^st^ day and returned to the normal range after that. This would suggest that, however Salmonella causes an increase in the amount of serum iron, but probiotics inhibit this mechanism. Geith *et al*. (2015) reported beneficial role of *Lactobacillus acidophilus* on the modification of acute phase parameters such as decreased fibrinogen, ESR, TIBC (total iron-binding capacity), UIBC (unsaturated iron-binding capacity), and ceruloplasmin and on the other hand increased albumin, total protein, iron, and transferrin saturation percentage during the *S*. Typhimurium infection in albino rats^4^.

### Inflammatory mediators determination

**Figure 1:**
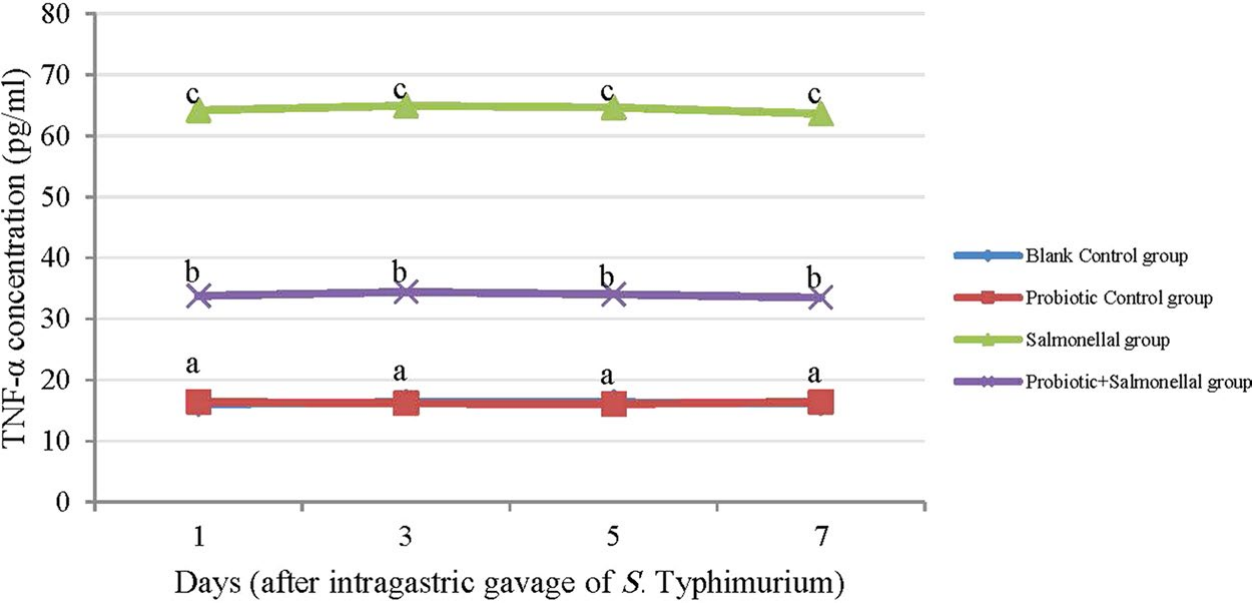
The amount of TNF-α (pg/ml) in serum samples of rats with different treatments in 1, 3, 5 and 7 days after gavaging Salmonella. Values are mean of 5 independent replicates. Blank Control group: Receiving water; Probiotic Control group: Receiving water containing 5 × 10^7^ spores/ml of *B. subtilis* and 5 × 10^7^ spores/ml of *B. coagulans*; Salmonella group: Receiving water and intragastric gavage of 1 × 10^9^ CFU of *S*. Typhimurium; Probiotic+Salmonella group: Receiving water containing 5 × 10^7^ spores/ml of *B. subtilis* and 5 × 10^7^ spores/ml of *B. coagulans* and intragastric gavage of 1 × 10^9^ CFU of *S*. Typhimurium. The line of Blank Control and Probiotic Control groups are overlapped. Different letters indicate significant differences between groups in each day (P < 0.05).

**Figure 2:**
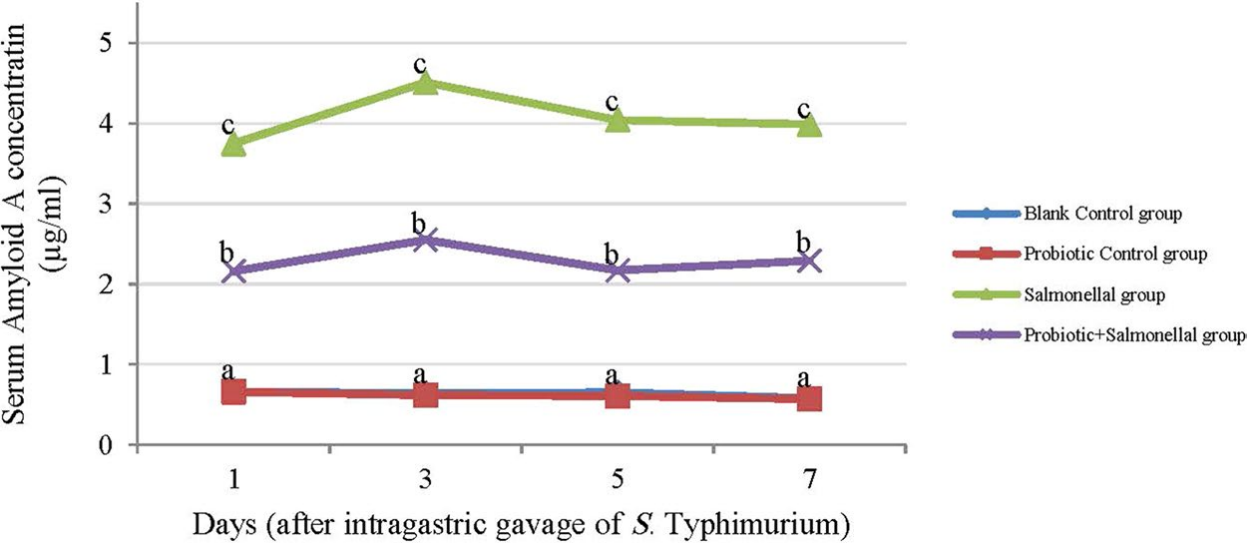
The amount of Serum Amyloid A (µg/ml) in serum samples of rats with different treatments in 1, 3, 5 and 7 days after gavaging Salmonella. Values are mean of 5 independent replicates. Blank Control group: Receiving water; Probiotic Control group: Receiving water containing 5 × 10^7^ spores/ml of *B. subtilis* and 5 × 10^7^ spores/ml of *B. coagulans*; Salmonella group: Receiving water and intragastric gavage of 1×10^9^ CFU of *S*. Typhimurium; Probiotic+Salmonella group: Receiving water containing 5 × 10^7^ spores/ml of *B. subtilis* and 5 × 10^7^ spores/ml of *B. coagulans* and intragastric gavage of 1 × 10^9^ CFU of *S*. Typhimurium. The line of Blank Control and Probiotic Control groups are overlapped. Different letters indicate significant differences between groups in each day (P < 0.05).

The level of inflammatory mediators, including TNF-α, serum amyloid A (SAA) and IL-10 in the Probiotic Control group and the Blank Control group was similar (*P* > 0.05). Salmonella infection significantly increased the level of TNF-α and SAA in all days of sampling (*P* < 0.05). With the Probiotic+Salmonella group, the level of these two inflammatory mediators significantly decreased compared to the Salmonella group (*P* < 0.05), but was still higher than the Blank Control and Probiotic Control groups (*P* < 0.05) (Figs. 1 and 2).

**Figure 3:**
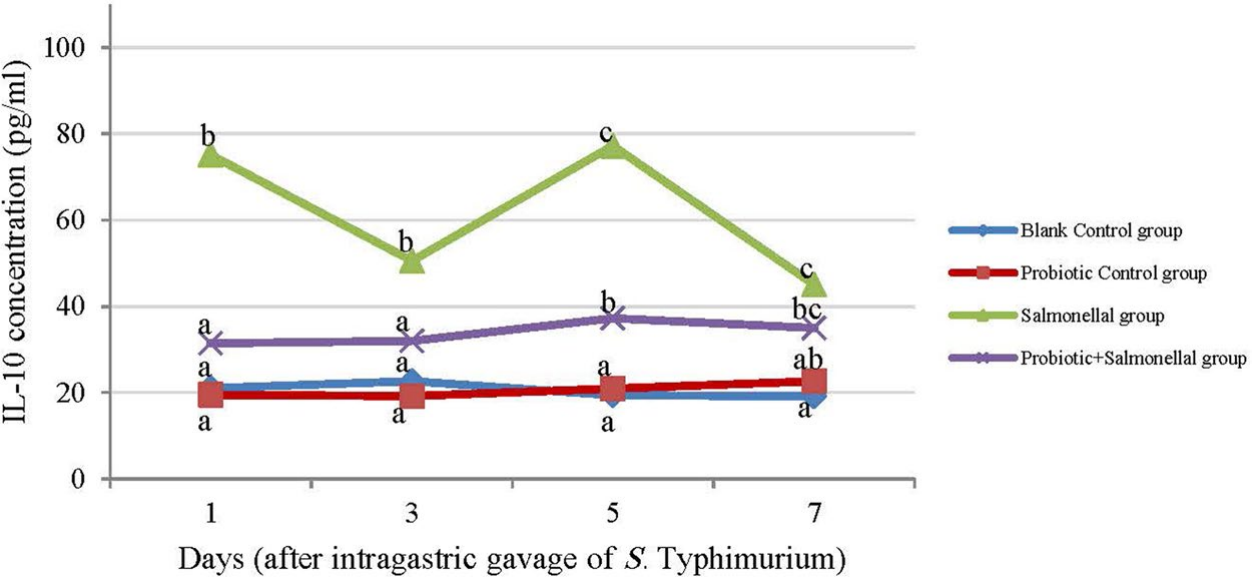
The amount of IL-10 (pg/ml) in serum samples of rats with different treatments in 1, 3, 5 and 7 days after gavaging Salmonella. Values are mean of 5 independent replicates. Blank Control group: Receiving water; Probiotic Control group: Receiving water containing 5 × 10^7^ spores/ml of *B. subtilis* and 5 × 10^7^ spores/ml of *B. coagulans*; Salmonella group: Receiving water and intragastric gavage of 1 × 10^9^ CFU of *S*. Typhimurium; Probiotic+Salmonella group: Receiving water containing 5 × 10^7^ spores/ml of *B. subtilis* and 5 × 10^7^ spores/ml of *B. coagulans* and intragastric gavage of 1 × 10^9^ CFU of *S*. Typhimurium. Different letters indicate significant differences between groups in each day (P < 0.05).

Salmonella infection significantly increased IL-10 levels in all days compared to the Blank Control group (*P* < 0.05), while probiotic consumption prevents the increase of IL-10 level up to 3^rd^ day after Salmonella gavaging. However, on day 5, the level of IL-10 increased significantly and decreased again on the 7^th^ day compared to the Blank Control group (Fig. 3). The correlation coefficient between the number of Salmonella in the ileum, cecum, feces, liver, spleen and mesenteric lymph nodes with the level of TNF-α was 0.39, 0.41, 0.37, 0.46, 0.61 and 0.62; with the level of SAA was 0.39, 0.43, 0.37, 0.43, 0.64 and 0.61; and with the level of IL-10 was 0.43, 0.51, 0.41, 0.34, 0.56 and 0.59, respectively (*P* < 0.05).

Systemic infection and bacterial colonization in the intestine induce SAA proteins in the liver^36^. TNF-α is produced early in the response to Lipopolysaccharide (LPS) and is believed to be a major mediator of the diverse pathophysiologic responses to LPS^37^. Also, IL-10 works as an essential molecule to allow *S*. Typhimurium infection by reducing the soluble inflammatory determinants that can activate both innate and adaptive immune responses that restrict *S*. Typhimurium dissemination^38^. In the current study, *S*. Typhimurium caused the increase in IL-10, TNF-α and SAA but *B. subtilis* and *B. coagulans* decrease these parameters. These probiotics reduce the number of Salmonella in the intestine and inhibit the colonization of this pathogen. Also, lower LPS levels are witnessed resulting in SAA, TNF-α and IL-10 showing lower changes in comparison to the Salmonella group. The decrease in the number of Salmonella in the ileum, cecum, feces, liver, spleen and mesenteric lymph nodes was accompanied with SAA, TNF-α and IL-10 reductions (*P* < 0.05). Feng *et al*. (2016) showed that *Lactobacillus plantarum* PZ01, *Lactobacillus salivarius* JM32 and *Pediococcus acidilactici* JH231 reduced the levels of lipopolysaccharide-induced TNF-α factor (LITAF), IL-1β, IL-6 and IL-12 in serum and increased the level of IL-10 in serum during a Salmonella infection in broiler chicks^5^.

### Total antioxidant capacity (TAC)

**Figure 4:**
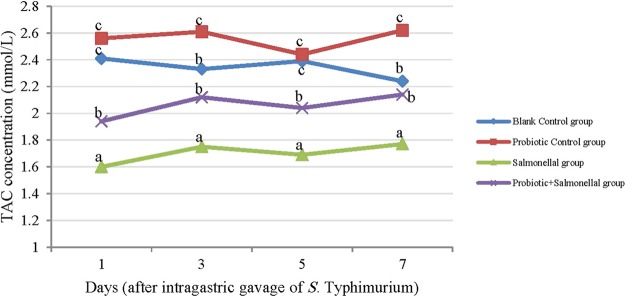
The amount of total antioxidant capacity (TAC) (mM/L) in serum samples of rats with different treatments in 1, 3, 5 and 7 days after gavaging Salmonella. Values are mean of 5 independent replicates. Blank Control group: Receiving water; Probiotic Control group: Receiving water containing 5 × 10^7^ spores/ml of *B. subtilis* and 5 × 10^7^ spores/ml of *B. coagulans*; Salmonella group: Receiving water and intragastric gavage of 1 × 10^9^ CFU of *S*. Typhimurium; Probiotic+Salmonella group: Receiving water containing 5 × 10^7^ spores/ml of *B. subtilis* and 5 × 10^7^ spores/ml of *B. coagulans* and intragastric gavage of 1 × 10^9^ CFU of *S*. Typhimurium. Different letters indicate significant differences between groups in each day (P < 0.05).

Salmonella infection reduced the amount of TAC significantly (*P* < 0.05), while the use of probiotics during exposure to Salmonella, increased the TAC (*P* < 0.05) (Fig. 4). The correlation coefficient between the number of Salmonella in the ileum, cecum, feces, liver, spleen and mesenteric lymph nodes with the amount of TAC was -0.36, -0.37, -0.34, -0.39, -0.43 and -0.48 in order (*P* < 0.05).

During the inflammatory processes, the phagocyte activation and bacterial products enhance the multicomponent flavoprotein NADPH oxidase, which catalyzes the production of high amounts of the superoxide anion radicals. As a result, the body requires adequate levels of antioxidant to avoid the reactive oxygen species harmful effects. Hence, antioxidants are necessary to regulate the reactions that release free radicals^39^. *S*. Typhimurium enhances oxidative stress. LPS of *S*. Typhimurium can induce H_2_O_2_ generation and a concomitant decline in TAC^40^. In this study, the TAC decreased more in the Salmonella group than Probiotic+Salmonella group. The increase in the number of Salmonella in the ileum, cecum, feces, liver, spleen and mesenteric lymph nodes was accompanied with TAC reduction (*P* < 0.05). This shows that probiotics reduce the destructive effects of Salmonella induced stress. Also, *B. subtilis* and *B. coagulans* as probiotics cause increases in TAC amounts in comparison to the Blank Control group. So these probiotics prepare the body for exposure to the pathogen and the stress that is caused by them.

### Measurement of malondialdehyde (MDA)

**Figure 5:**
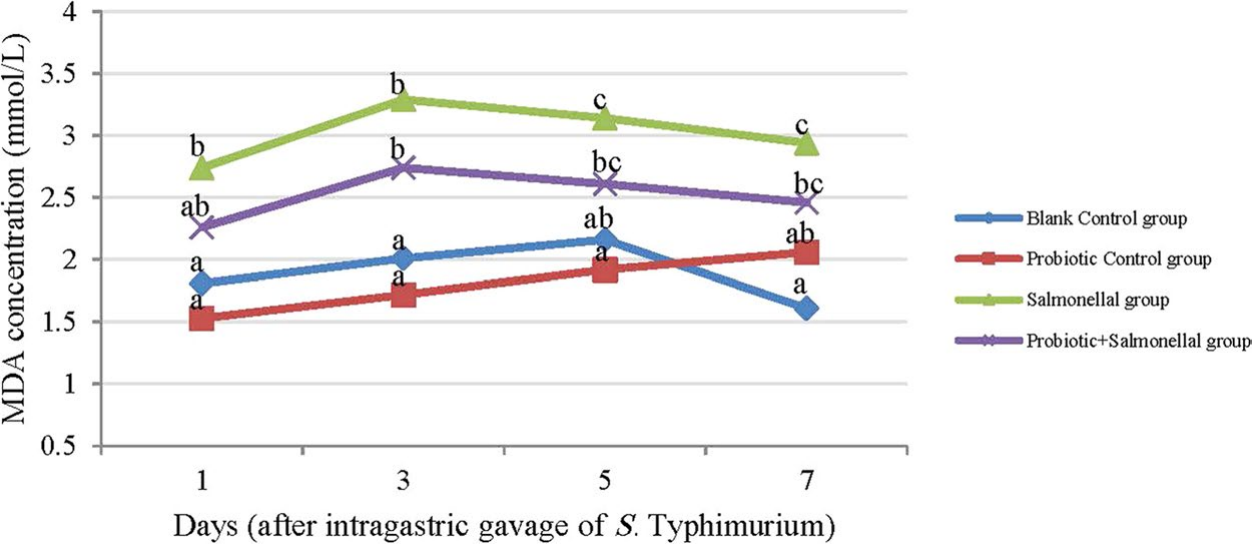
The amount of malondialdehyde (MDA) (mM/L) in serum samples of rats with different treatments in 1, 3, 5 and 7 days after gavaging Salmonella. Values are mean of 5 independent replicates. Blank Control group: Receiving water; Probiotic Control group: Receiving water containing 5×10^7^ spores/ml of *B. subtilis* and 5 × 10^7^ spores/ml of *B. coagulans*; Salmonella group: Receiving water and intragastric gavage of 1 × 10^9^ CFU of *S*. Typhimurium; Probiotic+Salmonella group: Receiving water containing 5 × 10^7^ spores/ml of *B. subtilis* and 5 × 10^7^ spores/ml of *B. coagulans* and intragastric gavage of 1 × 10^9^ CFU of *S*. Typhimurium. Different letters indicate significant differences between groups in each day (P < 0.05).

The amount of MDA during the sampling period was higher in the Salmonella and Probiotic+Salmonella groups compared to the Blank Control and Probiotic Control groups. Using probiotics during Salmonella infection, reduced the MDA, but the reduction was not significant (*P* > 0.05). The MDA content in the Probiotic Control group was similar to the Blank Control group (Fig. 5).

The correlation coefficient between the number of Salmonella in the ileum, cecum, feces, liver, spleen and mesenteric lymph nodes with the amount of MDA was 0.32, 0.32, 0.31, 0.31, 0.47 and 0.53 in order (*P* < 0.05). Malondialdehyde has been the most frequently used biomarker of lipid peroxidation and oxidative stress in many health problems^41^. MDA is one of the products that increased in Salmonella disease. The MDA in the Probiotic+Salmonella group has no significant difference with the Salmonella group. We can conclude that *B. subtilis* and *B. coagulans* as probiotics do not affect on MDA concentration. However, the correlation results showed a lesser number of Salmonella in the ileum, cecum, feces, liver, spleen and mesenteric lymph nodes was accompanied a lesser amount of MDA (*P* < 0.05).

## Conclusion

*B. subtilis* and *B. coagulans* as probiotics show no significant changes in serological parameters in comparison to the Blank Control group. However, if these probiotics used as a preventative measure against Salmonella infection, they compete with this pathogen in the intestine and enhance the mucosal immune system, delaying the infiltration of Salmonella into lymph nodes, spleen and liver, reduce the inflammatory mediators, decrease oxidative stress, and decrease any hematological and biochemical changes. The beneficial effects listed above, changes the parameters to return to normal ranges faster.

## Methods

### Animal ethics

All methods were performed in accordance with the relevant guidelines and regulations by the Ethical Committees of Shiraz University and approved by the governing body (1395/9234308).

### Preparation of probiotic bacteria

Spray dried spores of *B. subtilis* (PRM102) and *B. coagulans* (PRM101) in dextrose powder (1 × 10^11^ spores/gr) was donated by the Pardis Roshd Mehregan Company, Iran. To confirm the spores concentration of the probiotics, one gr of each powder was dissolved in 100 ml tap water (1 × 10^9^ each spores/ml) and heated at 80 °C for 15 min, to kill the vegetative cells, and surface plating after tenfold serial dilution was done.

### Preparation of challenged bacterium

In order to prepare the solution for intragastric gavage, a pure colony of *S*. Typhimurium (ATCC 14028) was cultured in 100 ml of Tryptic Soy Broth (TSB) (Merck, Germany) for 24 h at 37 °C. After incubation, it was centrifuged at 5,000 ×g for 10 min at 4 °C and then the pellet was resuspended in 50 ml physiological saline. The plating was performed for the suspension to confirm the concentration of 2 × 10^9^ CFU/ml^20^.

### Experimental design

In this experimental study, 80 Spargue-Dawley rats weighing 170-180 gr were procured from the Department of animal lab, Shiraz University of Medical Sciences, Iran. They were housed in plastic cages and kept under 12-hour light/ dark condition, a temperature of 20–25 °C, humidity of 50–60%, and free access to food (commercial standard pellets) and water for adaptation to the new environment.

After an acclimatization period of 1 week, they were randomly divided into four main groups, each containing five subgroups (four rats in each subgroup) and treated as follows:

1. Blank Control group: Receiving drinking water containing 0.1% dextrose for 4 weeks and intragastric gavage of 0.5 ml normal saline and 0.5 ml 6% (w/v) NaHCO_3_ (to neutralize the gastric acid) on day 22 of the study.
2. Probiotic Control group: Receiving drinking water containing 5 × 10^7^ spores/ml of *B. subtilis* and 5 ×10^7^ spores/ml of *B. coagulans* for 4 weeks and intragastric gavage of 0.5 ml normal saline and 0.5 ml 6% (w/v) NaHCO_3_ on day 22 of the study.
3. Salmonella group: Receiving drinking water containing 0.1% dextrose for 4 weeks and intragastric gavage of 1 × 10^9^ CFU of *S*. Typhimurium in 0.5 ml physiological saline and 0.5 ml 6% (w/v) NaHCO_3_ on day 22 of the study.
4. Probiotic+Salmonella group: Receiving drinking water containing 5 × 10^7^ spores/ml of *B. subtilis* and 5 × 10^7^ spores/ml of *B. coagulans* for 4 weeks and intragastric gavage of 1 × 10^9^ CFU of *S*. Typhimurium in 0.5 ml normal saline + 0.5 ml 6% (w/v) NaHCO_3_ on day 22 of the study.

The concentration of probiotic spores fed to the rats was determined based on our previous study^42^ and the concentration of challenged bacterium was determined based on Havelaar *et al*. (2001), Abdel Hamid *et al*. (2013) and Kim *et al*. (2013)^26,43,44^.

For confirmation that the rats are Salmonella-free, on days 0, 7, 14 and 21 of the study, fecal samples from each subgroup were taken and examined. One gr of pooled fecal sample from each subgroup was added into 9 ml lactose broth (Merck, Germany) and incubated at 37 °C for 24 h. After incubation, 1 ml of that was added to 10 ml Selenite Cystine broth (Merck, Germany) and in parallel, 0.1 ml was added to 10 ml Rappaport Vassiliadis broth (Merck, Germany). They were incubated at 37 °C and 42 °C, respectively, for 24 h. After which a loop of the selective enrichment broth was cultured on Xylose Lysine Deoxycholate agar (XLD agar, Merck, Germany) and Brilliant Green agar (BG agar, Merck, Germany). The plates were incubated at 37 °C for 24 h and checked for Salmonella colonies^14,45^.

### Sampling

On days 1, 3, 5 and 7 after intragastric gavage of *S*. Typhimurium, one rat was chosen randomly from each subgroup (replicated independently five times for each treatment group) and blood samples were obtained from the heart in an anesthetic state. After that, the rats were euthanized and their liver, spleen, mesenteric lymph nodes and intestines were removed under aseptic condition.

Blood samples were collected into tubes with and without anticoagulant (EDTA). The sera were separated by centrifugation of the blood sample without anticoagulant at 3,000 × g for 10 min and stored at -20 °C until used. The blood samples with anticoagulant were used for hematological parameters and differential WBC count.

### Enumeration of Salmonella

The liver, spleen, mesenteric lymph nodes, the content of ileum and cecum and about 2 gr of feces were weighed and transferred into stomacher bags along with 9 times sterile phosphate buffer saline (PBS, pH: 7.2). They were then homogenized in a stomacher (Seward Stomacher 400 Blender BA6021, UK) for 2 min and then tenfold serial dilution was made in PBS. The dilutions were then surface plated on BG agar and incubated at 37 °C for 48 h. This process was duplicated for all the cultured samples. The Salmonella enumeration was expressed as CFU/gr^5,46^. Also, on the first day of sampling, one gr of liver, spleen and mesenteric lymph nodes were separately crushed using a scalpel and added into 9 ml lactose broth (Merck, Germany) and incubated at 37 °C for 24 h. After incubation, 1 ml of that was added to 10 ml Selenite Cystine broth (Merck, Germany) and in parallel, 0.1 ml was added to 10 ml Rappaport Vassiliadis broth (Merck, Germany). They were incubated at 37 °C and 42 °C, respectively, for 24 h. Then a loop of the selective enrichment broth was cultured on XLD agar and BG agar. The plates were incubated at 37 °C for 24 h and checked for Salmonella colonies^14,45^.

### Measurement of hematological parameters

Hematological parameters including PCV, Hb, RBC, platelets and WBC concentration, were measured using cell blood counter (Celltac α MEK6550, Nihon Kohden Company, Japan).

A blood smear with Giemsa staining was prepared and differentiation of white blood cells into neutrophils, lymphocytes, monocytes, eosinophils, and basophils were done.

### Measurement of biochemical parameters

All biochemical parameters including, total protein, albumin, globulin, ALP, ALT, AST, glucose, LDH, creatine, BUN and total iron were measured by autoanalyzer (Alpha Classic AT++, Sanjesh Company, Iran).

### Determination of inflammatory mediators

SAA, TNF-α, and IL-10 levels were measured using a solid phase sandwich ELISA method (Rat ELISA Kits; Bioassay Technology Laboratory, Shanghai Crystal Day Biotech Company, LTC, China) and the absorption was read using an ELISA reader (Convergys ^®^EL-Reader 96×, Coelbe Company, Germany).

### Measurement of TAC

The commercial kit (ZellBio Com, GmbH Germany) was employed to measure TAC. At the end, a colour product of the chromogenic substrate (tetramethylbenzidine) appeared. The change in colour was measured colorimetrically at 450 nm and expressed as millimoles/ liter (mM/L).

### Measurement of MDA

The commercial kit (ZellBio Com, GmbH Germany) was employed to measure MDA and values were expressed as mM/L.

### Statistical analysis

The results were analyzed using analysis of variance and the statistical significance of differences between mean values was analyzed by Duncan’s multiple range tests. Pearson’s correlation coefficient test was applied to assess the correlation between variables. P-values less than 0.05 were considered statistically significant. The analysis was performed using Statistical Package for Social Sciences (SPSS) software (SPSS 16 for windows, SPSS Inc, Chicago, IL, USA).
